# Serum and Liver Tissue Metabonomic Study on Fatty Liver in Rats Induced by High-Fat Diet and Intervention Effects of Traditional Chinese Medicine Qushi Huayu Decoction

**DOI:** 10.1155/2017/6242697

**Published:** 2017-09-05

**Authors:** Xiao-jun Gou, Qin Feng, Lin-lin Fan, Jian Zhu, Yi-yang Hu

**Affiliations:** ^1^Central Laboratory, Baoshan District Hospital of Integrated Traditional Chinese and Western Medicine of Shanghai, Shanghai 201999, China; ^2^Institute of Liver Disease, Shuguang Hospital, Shanghai University of Traditional Chinese Medicine, Shanghai 201203, China; ^3^Department of Pharmacy, Zhongshan Hospital, Fudan University, Shanghai 200032, China; ^4^Department of Gastrointestinal Surgery, Ruijin Hospital, Shanghai Jiaotong University School of Medicine, Shanghai 200025, China

## Abstract

Qushi Huayu Decoction (QSHY), clinically derived, consists of five crude drugs, commonly used in treating fatty liver in a clinical setting. However, little is known about its metabolomics study. Herein, the serum and liver tissue metabolomics approach, based on gas chromatography coupled to spectrometry (GC/MS), was employed to evaluate the efficacy and the mechanism underlying QSHY in a rat model of high-fat diet-induced fatty liver. With pattern recognition analysis of serum and liver tissue metabolite profile, a clear separation of model group and control group was acquired for serum and liver tissue samples, respectively. The QSHY group showed a predisposition towards recovery mimicking the control group, which was in agreement with the biochemical alterations and histological results. 23 candidate biomarkers were identified in the serum and liver tissue samples that were utilized for exploring the underlying mechanism. The present study suggests that QSHY has significant anti-fatty liver effects on high-fat diet-induced fatty liver in rats, which might be attributed to regulating the dysfunction of beta-alanine metabolism, alanine, aspartate, and glutamate metabolism, glycine, serine, and threonine metabolism, pyruvate metabolism, and citrate cycle. Thus, metabolomics is a useful tool in the evaluation of the efficacy and elucidation of the mechanism underlying the complex traditional Chinese medicine prescriptions.

## 1. Introduction

Nonalcoholic fatty liver disease (NAFLD) is the most common cause of liver dysfunction, which represents a wide spectrum of metabolic abnormalities ranging from simple steatosis to nonalcoholic steatohepatitis (NASH) and advanced hepatic fibrosis as well as cirrhosis [1, 2]. It is strongly associated with the components of the metabolic syndromes including obesity, insulin resistance, dyslipidemia, and type 2 diabetes mellitus (T2DM) [3]. The prevalence of NAFLD in the general adult population has been estimated as 15–40% in Western countries and 9–40% of the Asian population [4]. NAFLD is under intensive focus and regarded as a public health concern. Currently, an established therapy for the treatment for NAFLD is lacking. However, lifestyle modification for weight loss and increased physical activity have been promoted as the primary treatment; nonetheless, long-term success in maintaining the lost weight has proven to be difficult. To date, no specific registered drug is available for the treatment of NAFLD [5]. Therefore, the prevention of fatty liver is an urgent prerequisite both theoretically and practically. Recently, the studies for developing new drugs focus on natural products with milder efficacy but also fewer side effects. Traditional Chinese medicine (TCM) has been practiced widely in China for thousands of years and is such a complementary and alternative therapeutic approach.

Qushi Huayu Decoction (QSHY), derived from clinical experience, consists of five crude drugs,* Herba Artemisiae Capillaris, Rhizoma Polygoni Cuspidati, Herba Hyperici Japonici, Rhizoma Curcumae Longae, and Gardenia Jasminoides Ellis*. Our previous study showed that QSHY had marked protective and therapeutic effects on fatty liver rat models induced by a high-fat diet or CCl_4_ combined with high-fat and low-protein diet, respectively. QSHY significantly reduced the liver triglyceride (TG) and free fatty acid (FFA) content and the level of serum tumor necrosis factor-alpha (TNF-*α*), improved liver steatosis, and ameliorated the inflammation [6, 7]. The major bioactive components of several single herbs, composed of QHSY, polygonin, rhein, and geniposide, have been shown to inhibit the progression of fatty liver [8–10]. Therefore, these constituents could contribute to our understanding of the therapeutic mechanism of QSHY. However, due to the complexity of interactions between the active components, the studies on the anti-fatty liver mechanism are challenging, accounting for the complex pathophysiology of fatty liver. Thus, exploring the anti-fatty liver mechanism underlying QSHY is rather challenging when using the conventional methods. Hence, novel approaches suitable for the complex system are in great demand and can improve the comprehensive evaluation of systemic clinical efficacy and mechanism of QSHY.

Metabolomics has been defined as “the quantitative measurement of the multiparametric response of living systems to the pathophysiological stimuli or genetic modification” [11]. It emphasizes the systematic characterization of the compositions of samples and focuses on the analysis of the entire pattern of low molecular weight compounds rather than individual metabolites. This research strategy is consistent with the integrity and systemic feature of TCM [12]. Nowadays, metabolomics is regarded as a potentially powerful application in some fields involving the discovery of novel potential biomarkers for diseases [13–15], evaluation of the efficacy of several herbal TCM prescriptions' efficacy [16–18], and assessment of the drug safety [19–21]. The common analytical tools for metabolomic studies comprise nuclear magnetic resonance spectroscopy (NMR), mass spectrometry (MS) combined with gas chromatography (GC/MS), liquid chromatography (LC/MS), and capillary electrophoresis (CE/MS). Among these, GC/MS that offers adequate separation of complex specimens, a high sensitivity, and resolution of metabolites combined with easily accessible NIST database has evolved as a popular and useful analytical technique in the study of metabolomics [22].

In the present study, a metabolomics method based on GC/MS with multivariate statistical techniques has been used to evaluate the efficacy of QSHY in a rat model of fatty liver induced by high-fat diet. In addition, the potential biomarkers related to the anti-fatty liver effect were identified and the underlying mechanism was explored. The clinical biochemistry and histological assessments were also carried out to ensure the success of the fatty liver model and investigate the anti-fatty liver effect of QSHY. Although QSHY-mediated anti-fatty liver is beyond the scope of this study, a metabolomics approach could serve as a promising scientific platform for therapeutic evaluation and the mechanistic study of TCM.

## 2. Materials and Methods

### 2.1. Chemicals

Methanol and 2-chlorophenylalanine were of analytical grade from China National Pharmaceutical Group Corporation (Shanghai, China). Heptadecanoic acid, N,O-bis(trimethylsilyl)trifluoroacetamide (BSTFA), and methoxyamine hydrochloride were obtained from Sigma-Aldrich (St. Louis, MO, USA). Commercial kits used for determining alanine aminotransferase (ALT), aspartate aminotransferase (AST), low-density lipoprotein cholesterol (LDL-C), high-density lipoprotein cholesterol (HDL-C), and triglyceride (TG) were obtained from Nanjing Jiancheng Institute of Biotechnology (Nanjing, China).

### 2.2. Preparation of QSHY

Qushi Huayu Decoction (QSHY) consists of the following five dried crude herbs,* Herba Artemisiae Capillaris*,* Rhizoma Polygoni Cuspidati*,* Herba Hyperici Japonici*,* Rhizoma Curcumae Longae*, and* Gardenia Jasminoides Ellis*, provided by the Shanghai Hua Yu Chinese Herbs Co., Ltd., China, and accredited by a pharmacognosist. The raw herbs were prepared in the ratio of 4 : 4 : 3 : 3 : 2.* Herba Artemisiae Capillaris*,* Rhizoma Polygoni Cuspidati*, and* Rhizoma Curcumae Longae* were extracted with ethanol two times, 1.5 h each time. The two ethanol extracting solutions were filtered and mixed.* Herba Hyperici Japonici* and* Gardenia Jasminoides Ellis* were decocted two times (1.5 h each time) in boiling water. The decoction was filtered, mixed, concentrated to a relative density, and purified by ethanol precipitation. Subsequently, the ethanol extraction and the water fraction were mixed and homogenized together and evaporated under vacuum at 40°C using a rotary evaporator. The concentration of the final stock solution of QSHY extract was adjusted to 0.93 g/crude herb/mL.

### 2.3. Experimental Animals

The study was carried out under the Guidelines for the Animal Experimentation of Shanghai University of Traditional Chinese Medicine (Shanghai, China), and the protocol was approved by the Animal Experiment Ethics Committee of Shanghai University of Traditional Chinese Medicine. Thirty male Sprague–Dawley (SD) rats (weighing 170 ± 20 g) were obtained from the Shanghai Experimental Animal Center of Chinese Academy of Sciences. Animal food was commercially obtained from the Shanghai Laboratory Animal Center (SLAC, Shanghai, China). All animals were maintained at 23-24°C and 60 ± 10% humidity on a 12/12 h light-dark cycle. The rats were fed with certified standard chow and tap water ad libitum for 1-week acclimation.

### 2.4. Animal Treatment

After 1-week acclimation, 30 rats were randomly assigned to three groups: (1) the control group were fed with a normal diet (13.8% fat, 60.5% carbohydrate, and 25.7% protein) for 8 weeks and given saline orally in the last 4 weeks daily, (2) the model group were fed with a high-fat diet (36.5% fat, 44.6% carbohydrate, and 18.9% protein) for 8 weeks and given saline orally in each of the last 4 weeks daily, and (3) QSHY were fed with a high-fat diet for 8 weeks and given 0.1 mL/kg/d QSHY orally for the last 4 weeks as described previously [23]. Each group included 10 rats and the body weights were monitored weekly throughout the experiment.

### 2.5. Sample Collection

All the rats were sacrificed by anesthesia with 2% sodium pentobarbital (3 mL/kg) after 8 weeks. The serum samples were collected from the abdominal aorta, centrifuged at 3000 rpm for 10 min, and stored at −80°C until further usage. The livers were weighed immediately and washed with normal cold saline. The samples from the right liver lobes were fixed in 10% neutral formalin for histological analysis. The samples from the left liver lobes were frozen at −80°C for subsequent experiments.

### 2.6. Analysis of Liver Function

The serum contents, including triglycerides (TG), low-density lipoprotein cholesterol (LDL-c), high-density lipoprotein cholesterol (HDL-c), alanine aminotransferase (ALT), and aspartate aminotransferase (AST), were measured using ELISA Kit according to the manufacturer's instructions.

### 2.7. Measurement of Liver Triglycerides

The TG content of the liver was measured as follows: 200 mg wet liver was homogenized in 3 mL ethanol-acetone (1 : 1) and maintained at 4°C for 12 h. Subsequently, the sample was centrifuged at 3000 rpm for 15 min, and the supernatant was collected for determination of the cholesterol content in the liver using commercial kits (Nanjing Jiancheng Institute of Biotechnology, China). The amount of TG in the liver was expressed as mg/g wet tissue.

### 2.8. Histological Assessment

A portion from each of the right lobes of the liver obtained from each rat was fixed in 10% neutral formalin, embedded in paraffin, and sliced into 5 *μ*m thick sections. The samples were stained with hematoxylin-eosin (H&E) and Sirius red, respectively [24].

### 2.9. Preparation of Metabolomic Samples

The serum samples were thawed at 4°C before analysis, and 100 *μ*L aliquots of blood were added to 400 *μ*L methanol for protein deposition. The mixture was vortexed for 1 min, followed by the addition of 60 *μ*L of 0.2 mg/mL 2-chlorophenylalanine and 60 *μ*L of 0.1 mg/mL heptadecanoic acid (internal standard). The mixture was vortexed for 1 min and centrifuged at 12,000 ×g, 4°C for 10 min. The supernatant was transferred to a centrifuge tube and the sample was concentrated to dryness by a vacuum centrifuge, and the dried extracts were methoximated in pyridine with 60 *μ*L of 15 mg/mL methoxyamine at 30°C for 2 h. Subsequently, the mixture was trimethylsilylated at 37°C for 1.5 h with 60 *μ*L BSTFA with 1% TMCS. The mixture was centrifuged at 12,000 ×g, 4°C for 10 min. Finally, the supernatant was added to the GC vial before GC/MS analysis.

100 mg liver tissue was added in a 2 mL tube containing 1000 *μ*L of methanol : water mixed solution (4 : 1, −20°C) and 5 balls. The mixture was placed in a high-flux tissue mill and ground at 70 Hz for 1 min, followed by the addition of 60 *μ*L of 0.2 mg/mL 2-chlorophenylalanine and 60 *μ*L of 0.1 mg/mL heptadecanoic acid (internal standard). The mixture was vortexed for 30 s, placed in an ultrasonic machine for 30 min at room temperature, placed on ice for 30 min, and centrifuged at 12000 ×g, 4°C, for 10 min. 800 *μ*L supernatant was transferred to a 1.5 mL tube, and the sample was concentrated by a vacuum centrifuge. The dried extract was methoximated in pyridine with 60 *μ*L of 15 mg/mL of methoxyamine at 30°C for 2 h. The mixture was subsequently trimethylsilylated at 37°C for 90 min with 60 *μ*L BSTFA with 1% TMCS. The mixture was centrifuged at 12,000 ×g at 4°C for 10 min. Finally, the supernatant was added to the detection bottles before GC/MS analysis.

### 2.10. GC/MS Analysis Injection

Each 1 *μ*L aliquot of the analytes was injected at a split ratio of 20 : 1 into an Agilent 6890N GC/5975B inert MSD (Agilent Technologies, Santa Clara, CA, USA). The separation was achieved on an HP-5MS capillary column (30 m × 250 *μ*m i.d., 0.25 *μ*m film thickness; 5% phenyl methylpolysiloxane bonded and crosslinked; Agilent J&W Scientific, Folsom, CA, USA). MS parameters: the injection temperature was set at 280°C, the interface temperature was set at 150°C, and the ion source was adjusted to 250°C. The GC oven temperature was maintained at 70°C for 2 min and then ramped at 10°C/min to 300°C for 5 min. Helium was used as the carrier gas at a flow rate of 1 mL/min. The electron energy was 70 eV, and the detection was conducted in full scan mode (*m*/*z* 35–780). The solvent delay was 5 min.

### 2.11. Pattern Recognition Analysis and Statistical Analysis

The unprocessed GC/MS raw files were converted to NetCDF format via GC/MS with its function and then processed by the XCMS toolbox (https://xcmsonline.scripps.edu/) with default settings to carry out baseline correction, peak discrimination and alignment, and the correction of the retention time. The resulting table (TSV file) was exported into Microsoft Excel. All data were normalized to the total sum of spectrum prior to multivariate analyses. The data were analyzed by pattern recognition with multivariate statistical analysis tools. SIMCA-P11.5 software package (Umetrics AB, Umea, Sweden) was utilized for principal component analysis (PCA) and partial least-squares discriminant analysis (PLS-DA). Additionally, unpaired Student's *t*-test was employed to evaluate the significant difference if the discriminant score values or the concentrations of the differential metabolites obtained from PLS-DA model data were statistically significant between the model and control groups. The concentrations of the significantly altered metabolites were represented as their relative areas (divided by the area of internal standard).

The statistical analyses for the body weight and blood parameters were performed using the Statistical Package for SPSS 19.0 (SPSS, Chicago, USA). The quantitative data were expressed as means ± SD, and the statistical significance among the groups was analyzed by one-way analysis of variance using the Student–Newman–Keuls test. *P* < 0.05 was considered as statistically significant.

## 3. Results

### 3.1. Body Weight

The initial body weight did not differ significantly in the 3 groups. After 8 weeks, the model group developed significantly heavy body as compared to the control group rats (*P* < 0.05), while the body weight of the QSHY group rats decreased gradually and displayed no significant difference as compared to the model group rats (Table 1).

### 3.2. Blood ALT, AST, TG, and LDL-c Concentration

At the end of the administration, the serum ALT, AST, TG, and LDL-c levels increased significantly in the model group rats as compared to the control groups (*P* < 0.01). QSHY markedly lowered the ALT, AST, TG, and LDL-C levels (*P* < 0.05 or *P* < 0.01) as compared to the model group, respectively. No significant changes were observed in the serum HDL-c between the control and model groups (Table 1).

### 3.3. Hepatic Levels of TG

As shown in Table 1, after 8 weeks of high-fat diet, the rats in the model group exhibited increased TG level as compared to control group rats (*P* < 0.01). The treatment with QSHY decreased the levels of these markers as compared to the model group rats (*P* < 0.01).

### 3.4. Histological Examination of the Liver

**Figure 1:**
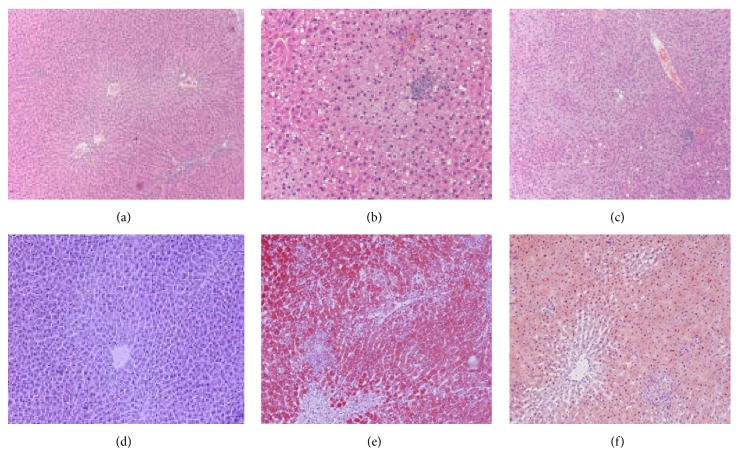
Liver sections showing steatosis and inflammation with H&E staining (×200) and Oil Red staining (×200). H&E staining from (a) control group, (b) model group, and (c) QSHY group. Oil Red staining (×200) from (d) control group, (e) model group, and (f) QSHY group.

The histological changes in the liver were examined in H&E-stained sections. The liver sample of the model rats presented a developing degeneration of hepatocytes with abundant fat deposition, inflammatory infiltration, significant hepatocyte ballooning, and a single large vacuole within the cytoplasm of liver cells. In the QSHY group rats, the histological characteristics of the fatty liver were persistent; however, the symptoms were alleviated. No histological signs were found in the control group rats (Figures 1(a)–1(c)). The histological examination with Oil Red staining showed that the liver cells were swollen and rounded, with unequal cellular staining; the liver cells around the central vein stained darker in the model group. In the QSHY group, the histological characteristics of fatty liver were observed; however, these symptoms were alleviated. No histological signs were found in the rats from the control group (Figures 1(d)–1(f)). The above phenomena manifested that fatty liver model induced by high-fat diet in rats was established successfully and that the histological condition improved with administration of QSHY.

### 3.5. Metabolomics Study

#### 3.5.1. GC/MS Spectra of the Three Groups

**Figure 2:**
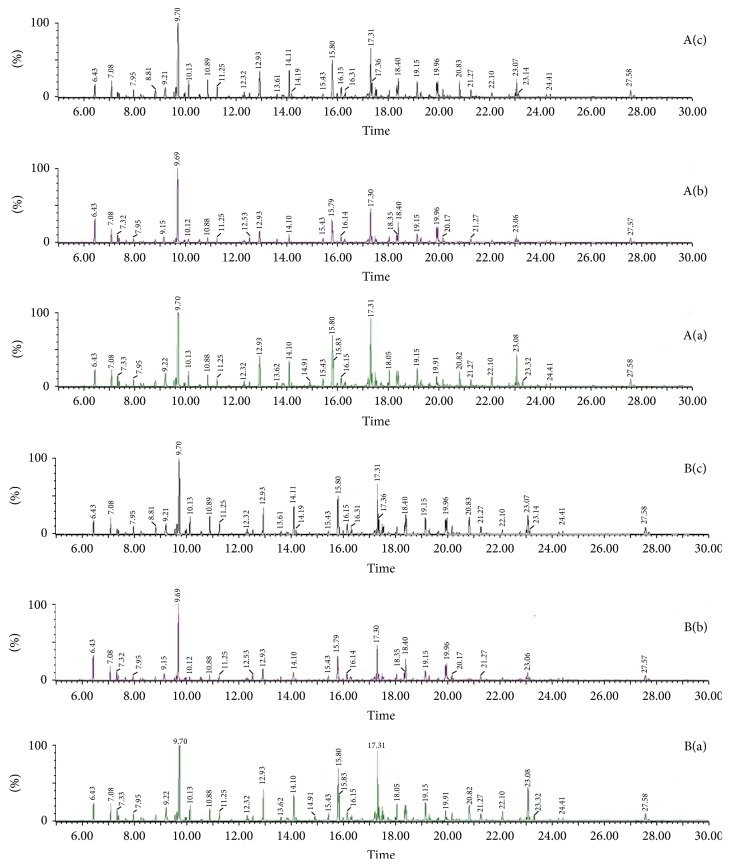
Typical GC/MS spectra of samples from serum ((A)(a), (A)(b), and (A)(c)) and liver tissue ((B)(a), (B)(b), and (B)(c)). (a) Control group, (b) model group, and (c) QSHY group.

A typical GC/MS total ion current (TIC) chromatogram of serum and liver tissue samples from the control, model, and QSHY groups was illustrated in Figure 2: serum ((A)(a), (A)(b), and (A)(c)) and liver tissue ((B)(a), (B)(b), and (B)(c)), respectively. A visual inspection of the spectra revealed some obvious differences; however, the complexity of GC/MS spectra interfered with a further comparison between the classes. In order to illustrate the differences in the metabolic profiles, GC/MS spectra were further pretreated, and a pattern recognition analysis was carried out.

#### 3.5.2. Analysis of Metabolic Profiles and Identification of Potential Biomarkers

In this study, a typical GC/MS total ion current (TIC) chromatogram of serum and liver tissue samples from the control, model, and QSHY groups was illustrated in Figure 2. Subtle changes could be found using a pattern recognition method, such as PCA and PLS-DA. PCA and PLS-DA are the two widely used pattern recognition methods for obtaining information on classification and identifying the metabolites. PCA, an unsupervised method, is applied as the first step in the separation procedure to filter out the noise and reduce the dimension of data among the observations. PLS-DA, a supervised method, with a principle similar to that of PCA, is used to enhance the classification performance [12]. The PCA for serum and liver tissue samples shows a poor separation between the control and model groups (data not shown).

**Figure 3:**
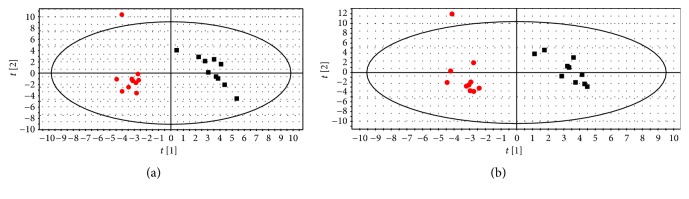
Score plot of control group (■) and model group (●) from a PLS-DA model of serum (a) and liver tissue (b) samples.

In order to improve the classification of the model and control groups, a PLS-DA model was established for the serum and liver tissue samples. As observed from Figures 3(a) and 3(b), the samples in the model and control groups of serum and liver tissue were separated. The parameters *R*2*X*, *R*2*Y*, and *Q*2*Y* of the model serum samples were 0.42, 0.96, and 0.82, respectively, and those of the liver tissue samples were 0.61, 0.98, and 0.73, respectively, which indicates that the metabolic profile of the model group rats is different from the control group rats.

Based on the result of PLS-DA score plot for serum and liver tissue samples and according to the parameter VIP (variable importance in the projection) [25], the *P* value of Student's *t*-test was <0.05. 23 endogenous metabolites in the serum and liver tissue samples were considered as potential biomarkers related to the group separation. Additionally, potential biomarkers were identified using the reference compounds available and the commercial compound libraries, NIST. Among those 23 potential biomarkers, 16 were commonly found in serum and liver tissue samples. Moreover, the concentration of 16 biomarkers was consistent in both the serum and liver tissue samples of the model group. The results were summarized in Table 2.

#### 3.5.3. The Influence of QSHY on the Metabolic Profiles of High-Fat Diet Rats

**Figure 4:**
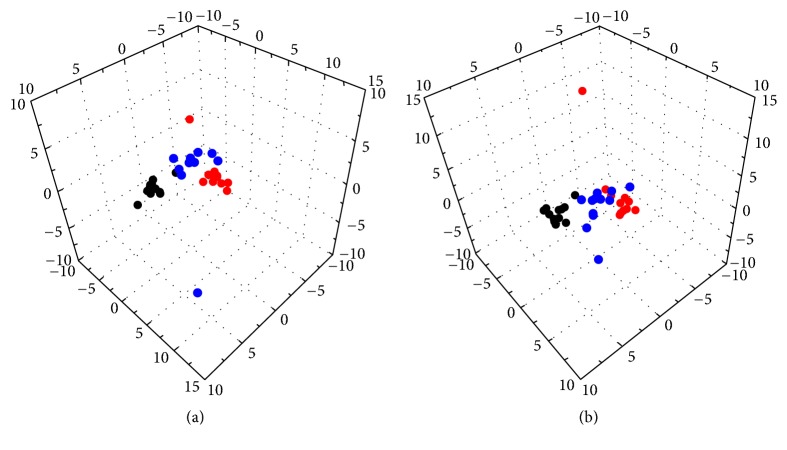
Score plot of 3D-PLS-DA model obtained from the control group (black bullet), model group (red bullet), and QSHY group (blue bullet) of serum (a) and liver tissue (b) samples.

In order to evaluate the influence of QSHY on the metabolic profiles of high-fat diet rats in serum and liver tissue samples, a three-dimensional PLS-DA scores' plot was constructed to illustrate the general variation between the control and model groups with QSHY intervention, respectively. Figures 4(a) and 4(b) for serum and liver tissue showed that, in the scores plot, the model and control groups could be distinctly separated. The results indicated that the metabolic pattern of the serum and liver tissue was significantly altered in the high-fat diet-treated model group. The PLS-DA scores plot also demonstrated that the QSHY-treated group was located between the model and control groups, and closer to the control group, implying that QSHY effectively prevented the progression of fatty liver and regulated the perturbed metabolism.

We also found that the mean level of the 17 metabolites showed a normal predisposition at different degrees after administration of QSHY (Table 2). Among these biomarkers, 17 metabolites in the QSHY group were completely reversed to the levels of that in the control group (Table 2). Therefore, it can be speculated that, after the administration of QSHY, the concentrations of these biomarkers, which were altered in the model group rats, had the tendency to return to that similar to the control group. Combined with the results of the biochemical parameters and histological examination, the above evidence indicated that QSHY exerted distinct anti-fatty liver effect. Thus, these biomarkers might explain the mechanisms underlying QSHY.

## 4. Discussion

In the present study, the histological findings confirmed that the high-fat diet led to the development of NAFLD in rats. The high-fat diet supplemented with QSHY markedly reduced the TG content in the liver tissue accompanied by a decrease in the serum levels of ALT, AST, TG, and LDL. QSHY lowered the body weight in high-fat diet rats and ameliorated the histological features of high-fat diet-treated rats. Together, these results suggested that the intake of QSHY may be valuable in preventing and improving the fatty liver induced by high-fat diet.

In addition to the biochemical and histological effects of QSHY, the metabolic pattern induced by high-fat diet and its influence based on GC/MS coupled with the pattern recognition were also investigated. QSHY was confirmed to affect the metabolic pattern of the high-fat diet-treated model. The QSHY-treated group exhibited a tendency of recovering to a healthy state. The concentration of 17 endogenous metabolites in the serum and liver tissue samples was significantly affected by the QSHY treatment, and these perturbations could be partially reversed by QSHY intervention. Thus, it could be inferred that these significantly altered metabolites were involved in the anti-fatty liver mechanism underlying QSHY.

**Figure 5:**
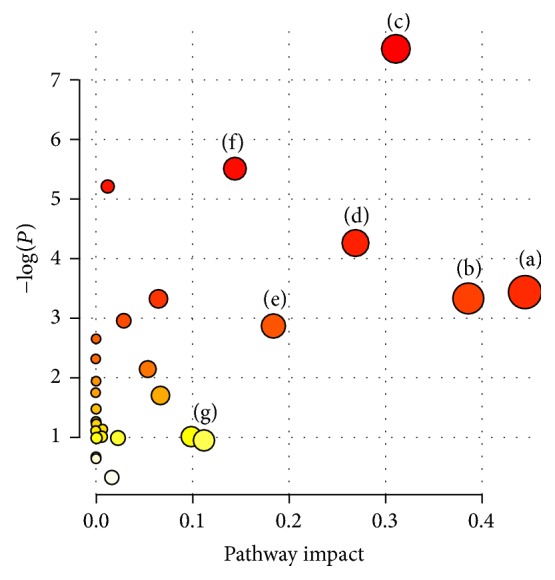
Summary of pathway analysis. (a) beta-Alanine metabolism, (b) glycerolipid metabolism, (c) alanine, aspartate, and glutamate metabolism, (d) glycine, serine, and threonine metabolism, (e) pyruvate metabolism and citrate cycle, (f) citrate cycle, and (g) inositol phosphate metabolism. Identification of the network pathway by MetPA software.

**Figure 6:**
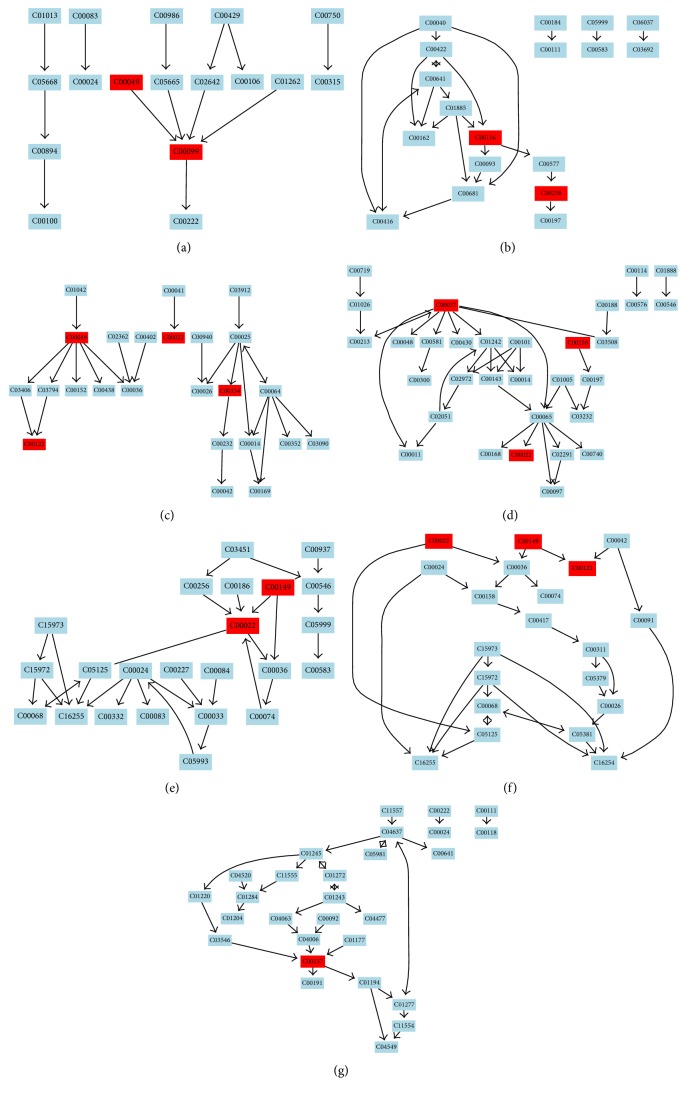
(a) beta-Alanine metabolism, (b) glycerolipid metabolism, (c) alanine, aspartate, and glutamate metabolism, (d) glycine, serine, and threonine metabolism, (e) pyruvate metabolism, (f) citrate cycle, and (g) inositol phosphate metabolism. The maps were generated using the reference map by KEGG (http://www.genome.jp/kegg/).

In order to further investigate the high-fat diet-induced liver fatty metabolomic profiling, 23 potential biomarkers related to group separation were imported to the online system, MetaboAnalyst (Figure 5). Previously, it has been postulated that a change occurring at a critical location in the network could trigger a severe impact compared to a change on the edge or a relatively isolated location [26]. In the present study, the impact threshold was set to 0.10. Any pathway above this threshold was classified as a potential target pathway. The top 7 metabolic pathways included beta-alanine metabolism (Figure 6(a)), glycerolipid metabolism (Figure 6(b)), alanine, aspartate, and glutamate metabolism (Figure 6(c)), glycine, serine, and threonine metabolism (Figure 6(d)), pyruvate metabolism (Figure 6(e)), citrate cycle (TCA cycle) (Figure 6(f)), and inositol phosphate metabolism (Figure 6(g)).

**Figure 7:**
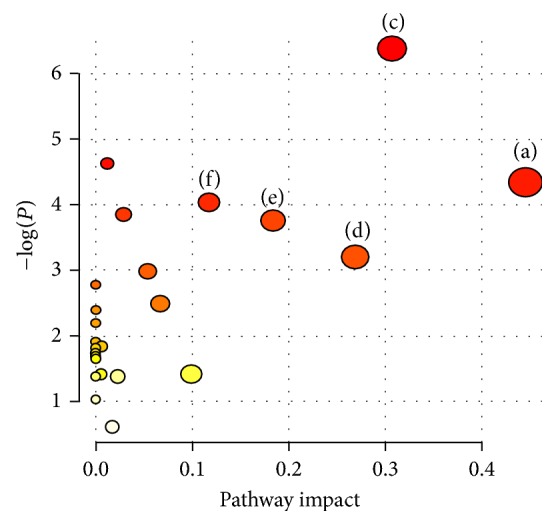
Summary of pathway analysis. (a) beta-Alanine metabolism, (c) alanine, aspartate, and glutamate metabolism, (d) glycine, serine, and threonine metabolism, (e) pyruvate metabolism, and (f) citrate cycle.

Drug targets are identified as key molecules involved in a specific metabolic or signaling pathway in a disease [27]. In this study, the metabolic changes associated with fatty liver in the QSHY-treated group were analyzed. Compared to the model group, the reversal trend of 17 different metabolites in the serum and liver tissue samples of the QSHY-treated group reached healthy levels (Table 2). According to the MetPA analysis (Figure 7), the beta-alanine metabolism, alanine, aspartate, and glutamate metabolism, glycine, serine, and threonine metabolism, pyruvate metabolism, and citrate cycle were potential targets for the design of the QSHY drug.

Evaluation of the metabolites at higher levels, from the pathway to network, allows understanding the physiological properties of the metabolites that affect the biological states. Metabolites that exert a significant effect on the relevant pathway (Figure 6) play a major role in the pathogenesis and possible complications of the disease [28]. In this study, alanine, aspartate, and pyruvate content in the blood of the model group rats decreased, and the pyruvate content in the liver homogenate of the model group rats decreased as compared to the control group rats; these changes indicated that the glycogenic oxyacids were converted to carbohydrates. This might be because the long-term high-fat diet led to energy metabolism disorders in rats, which affected gluconeogenesis and tricarboxylic acid cycle [29]. The concentrations of malic acid and fumaric acid in the liver homogenate of rats in the model group were decreased; these served as the intermediate substances in the tricarboxylic acid cycle, thereby resulting in the fact that the high-fat diet affected the tricarboxylic acid cycle in rats. The levels of alanine, aspartate, pyruvate, malic acid, and fumaric acid were upregulated by QSHY intervention, which suggested that QSHY intervened beta-alanine metabolism, alanine, aspartate, and glutamate metabolism, pyruvate metabolism, and citrate cycle that might be correlated to the therapeutic mechanism of QSHY (Figure 7).

In the model group, the glycine level was significantly decreased as compared to the control group (Table 2). Glycine is one of the three amino acids in GSH biosynthesis. GSH is a major antioxidant, which quenches the endogenous oxidant species and attacks the exogenous oxidative stress [30], and increased oxidative stress can act as a major molecular mechanism underlying the high-fat diet-induced fatty liver [31]. Glycine harbors the potential to act as a hepatospecific antioxidant to reduce the oxidant and cytokine production by Kupffer cells and promote the hepatic fatty acid oxidation [32]. Furthermore, glycine can synthesize serine; threonine is catalyzed by aldolase into glycine. Serine and threonine are both ketogenic amino acids, and a close relationship exists between metabolic disorders with respect to the metabolism of amino acids and the occurrence and development of fatty liver as well as other metabolic diseases. A long-term ketogenic diet can transform the body's amino acid metabolism and improve obesity as well as steatohepatitis [33]. Thus, we speculate that the decrease in glycine is due to the dysfunction of glycine, serine, and threonine metabolism (Figure 6(d)). According to the current study, glycine was decreased in high-fat diet-treated model rats and upregulated in QSHY group after QSHY treatment as compared to the model group rats. QSHY alleviated the consequences of fatty liver that may be associated with abnormalities in glycine, serine, and threonine metabolism (Figure 7).

In the current study, the alterations in the levels of docosahexaenoic acid, tetradecanoic acid, and oleic acid in QSHY-treated fatty liver rats were observed. NAFLD patients were reported to have high levels of saturated (SFA) and low levels of polyunsaturated fatty acids (PUFA) [34–36]. The intake of SFA is associated with a high level of insulin resistance and could lead to increased hepatic synthesis of TG. The reduction in hepatic TG accumulation might prevent the progression from steatosis to NAFLD [37]. Docosahexaenoic acid (DHA) is a member of the family of long-chain n-3 PUFA. Some studies have shown that dietary PUFA affected the levels of hepatic TG, insulin resistance, and inflammation [38]. Some studies have recently demonstrated that the supplemental n-3 fatty acids have potential therapeutic effects in human NAFLD [39]. These results suggested that high-fat diet affected the glycerolipid metabolism (Figure 6(b)). In this study, a decreased level of docosahexaenoic acid and elevated levels of tetradecanoic acid and oleic acid were observed in the model group as compared to the control group; the abnormalities were revealed in the QSHY group. Moreover, the high-fat diet supplemented with QSHY markedly reduced the TG content in the liver tissue. These results were in agreement with the several reports mentioned above, and QSHY had obvious anti-fatty liver effect by regulating the perturbations of fatty acids metabolism.

Inositol is a key metabolite of phosphoinositide metabolism (Figure 6(g)). Phosphoinositides have been shown to be crucial intracellular second messengers in the regulation of diverse physiological events regulated by the phosphorylation status [40] and dysregulated inositol phosphate formation as well as metabolism; these are associated with various pathophysiological conditions [41]. In the present study, inositol was significantly decreased in the model group as compared to the control group. The QSHY intervention of high-fat diet-treated rats showed a health-promoting state of the level of inositol. Thus, QSHY could putatively exert a major metabolic effect on inositol phosphate metabolism.

## 5. Conclusion

In the current study, a metabolomics approach based on GC/MS has been developed to establish the metabolomic profiles of serum and liver tissue in rats in order to investigate the anti-fatty liver effect of QSHY and its underlying mechanism. 23 endogenous metabolites in the serum and liver tissue samples were considered as potential biomarkers for the separation of the groups. Combined with the result of the assay evaluating the physiological parameters and the histology, the changes in the serum and liver metabolites suggested that the disorders of beta-alanine metabolism, alanine, aspartate, and glutamate metabolism, glycine, serine, and threonine metabolism, pyruvate metabolism, and citrate cycle are related to fatty liver induced by high-fat diet and the potential effect of QSHY on some of the above metabolic pathways. Furthermore, the current study demonstrated that GC/MS metabolomic profiling was found to be an optimal platform for evaluating the efficacy and the elucidation of the mechanism underlying the traditional Chinese medicines. Therefore, further studies are essential for substantiating the conclusions. Owing to the limited time and the economic difficulty, we studied only the samples of serum and liver tissue and did not observe a time-dependent dynamic change in the metabolomic profile. Thus, further studies will focus on collecting and analyzing the dynamic changes in the metabolomic profile of urine samples in order to explain mechanisms underlying the activities of QSHY.

## Figures and Tables

**Table 1:** Body weight and biochemical parameters from animals of different groups (mean ± SD, *n* = 10).

Item	Control group	Model group	QSHY group
Body weight (g)			
Initial	180.51 ± 6.12	182.86 ± 4.05	181.29 ± 5.76
Final	433.07 ± 36.28	469.07 ± 31.43^*∗*^	457.09 ± 44.65
Liver			
TG (mg/g)	1.14 ± 0.44	4.73 ± 1.35^*∗∗*^	3.51 ± 0.89^##^
Serum			
TG (mmol/L)	6.46 ± 0.58	7.30 ± 0.76^*∗∗*^	6.25 ± 0.67^##^
HDL-c (ng/L)	34.32 ± 9.19	31.13 ± 7.93	33.26 ± 8.32
LDL-c (ng/L)	74.05 ± 5.49	94.25 ± 8.43^*∗∗*^	83.36 ± 7.36^##^
ALT (U/L)	11.12 ± 1.89	13.88 ± 2.52^*∗∗*^	10.78 ± 2.16^#^
AST (U/L)	34.36 ± 4.16	40.31 ± 5.44^*∗∗*^	33.61 ± 4.77^##^

TG: triglycerides; HDL: high-density lipoprotein; LDL: low-density lipoprotein; ALT: alanine aminotransferase; AST: aspartate aminotransferase; significant differences compared to the control group (^*∗*^*P* < 0.05, ^*∗∗*^*P* < 0.01); significant differences compared to the model group (^#^*P* < 0.05, ^##^*P* < 0.01).

**Table 2:** Summary of potential biomarkers.

	Metabolites	Model group^a^	QSHY group^b^
Serum	Tetradecanoic acid	↑*∗∗*	↓
	Oleic acid	↑*∗∗*	↓
	Glycerol	↑*∗∗*	↓
	Glycine	↓*∗∗*	↑##
	Homoserine	↓*∗∗*	↑##
	Pyruvic acid	↓*∗∗*	↑#
	Docosahexaenoic acid	↓*∗∗*	↑
	Ribose	↓*∗∗*	↑
	Adenine	↓*∗∗*	↑#
	beta-Alanine	↓*∗∗*	↑##
	N-Acetylglutamic acid	↓*∗∗*	↑##
	Myo-inositol	↓*∗∗*	↑
	3-Phosphoglyceric acid	↓*∗*	↑
	Fumaric acid	↓*∗*	↑
	Malic acid	↓*∗*	↑#
	Adenosine	↓*∗*	↑#
	Glutaric acid	↓*∗*	↑#
	Glyceric acid	↓*∗*	↑
	Glycerol-2-phosphate	↓*∗*	↑#
	Aspartic acid	↓*∗*	↑#

Liver tissue	Tetradecanoic acid	↑*∗∗*	↓
	Oleic acid	↑*∗∗*	↓
	Glycerol	↑*∗∗*	↓
	Glycine	↓*∗∗*	↑##
	Cholesterol	↑*∗∗*	↓##
	N-Acetylglutamic acid	↓*∗∗*	↑##
	Campesterol	↓*∗∗*	↑##
	Glutaric acid	↓*∗∗*	↑##
	4-Aminobutyric acid	↓*∗*	↑##
	Adenosine	↓*∗*	↑#
	Adenine	↓*∗∗*	↑#
	Pyruvic acid	↓*∗∗*	↑#
	Glycerol-2-phosphate	↓*∗*	↑#
	Ribose	↓*∗∗*	↑#
	Docosahexaenoic acid	↓*∗∗*	↑
	Myo-inositol	↓*∗*	↑
	3-Phosphoglyceric acid	↓*∗*	↑
	Malic acid	↓*∗*	↑
	Fumaric acid	↓*∗*	↑

The up or down arrows represent the relatively increased or decreased levels of the metabolites in model group or QSHY group, respectively. ^a^Compared to the control group. ^b^Compared to the model group. ^*∗∗*^*P* < 0.01, ^*∗*^*P* < 0.05, ^##^*P* < 0.01, and ^#^*P* < 0.05.
